# One-year changes in brain microstructure differentiate preclinical Huntington's disease stages

**DOI:** 10.1016/j.nicl.2019.102099

**Published:** 2019-12-03

**Authors:** Chris Patrick Pflanz, Marina Charquero-Ballester, D.S. Adnan Majid, Anderson M. Winkler, Emmanuel Vallée, Adam R. Aron, Mark Jenkinson, Gwenaëlle Douaud

**Affiliations:** a Oxford Centre for Functional MRI of the Brain (FMRIB), Wellcome Centre for Integrative Neuroimaging, Nuffield Department of Clinical Neurosciences, University of Oxford, UK; b Department of Psychiatry, University of Oxford, UK; c Department of Psychology, University of California, San Diego (UCSD), San Diego, California, USA

**Keywords:** Preclinical Huntington's disease, Longitudinal, Microstructure, Corpus callosum, Basal ganglia, Diffusion imaging

## Abstract

**Objective:**

To determine whether brain imaging markers of tissue microstructure can detect the effect of disease progression across the preclinical stages of Huntington's disease.

**Methods:**

Longitudinal microstructural changes in diffusion imaging metrics (mean diffusivity and fractional anisotropy) were investigated in participants with presymptomatic Huntington's disease (*N* = 35) stratified into three preclinical subgroups according to their estimated time until onset of symptoms, compared with age- and gender-matched healthy controls (*N* = 19) over a 1y period.

**Results:**

Significant differences were found over the four groups in change of mean diffusivity in the posterior basal ganglia and the splenium of the corpus callosum. This overall effect was driven by significant differences between the group far-from-onset (FAR) of symptoms and the groups midway- (MID) and near-the-onset (NEAR) of symptoms. In particular, an initial decrease of mean diffusivity in the FAR group was followed by a subsequent increase in groups closer to onset of symptoms. The seemingly counter-intuitive decrease of mean diffusivity in the group furthest from onset of symptoms might be an early indicator of neuroinflammatory process preceding the neurodegenerative phase. In contrast, the only clinical measure that was able to capture a difference in 1y changes between the preclinical stages was the UHDRS confidence in motor score.

**Conclusions:**

With sensitivity to longitudinal changes in brain microstructure within and between preclinical stages, and potential differential response to distinct pathophysiological mechanisms, diffusion imaging is a promising state marker for monitoring treatment response and identifying the optimal therapeutic window of opportunity in preclinical Huntington's disease.

## Highlights

• Differences in preHD in one-year MD change in posterior basal ganglia and CC splenium.
• Non-monotonic effect driven by MD decrease in FAR group and increase in MID/NEAR.
• Only 1 clinical measure shows difference in 1y change between preclinical stages.
• Diffusion imaging may detect early signs of inflammation preceding degeneration.

## 1 Introduction

Huntington's disease (HD) is an autosomal dominant inherited, hyperkinetic-hypotonic movement disorder, caused by a polyglutamine-expansion. HD is considered to be a model neurodegenerative disorder, as it is amenable to predictive genetic testing with estimation of years to the onset of symptoms, thereby enabling the entire natural history of neurodegeneration to be studied from a presymptomatic stage at 100% risk of conversion to the disease (Ross et al., 2014).

Atrophy of the neostriatum, and more generally alterations in grey matter (GM) and white matter (WM) macrostructure, can be detected as much as 15y before the predicted onset of the disease using non-invasive neuroimaging techniques (Paulsen et al., 2006; Aylward et al., 2011). These findings suggest that clinical diagnosis of HD is not the start, but rather the endpoint of a series of pathophysiological alterations in the cerebral neural tissue. At the time of motor diagnosis, large parts of neuronal tissue are already irreversibly lost. Hence, there is a strong need for an early, prognostic state biomarker to monitor progression of the disease and proximity to the onset of the disease above and beyond genetic information, and to ensure that the optimal timeframe for disease modifying, neuroprotective therapy is not missed (Weir et al., 2011).

Striatal volume loss, as quantified using structural MRI, is a reliable biomarker of disease stage and progression that show longitudinal changes in preclinical HD (preHD) (Aylward et al., 2011; Majid et al., 2011a; Majid et al., 2013). However, these structural changes are inherently monotonic over the course of the neurodegenerative disease – with a progressive decrease in volume – making it difficult to capture differential effects *across* various presymptomatic stages of the disease.

This caveat might be overcome using diffusion MRI, a technique sensitive to microstructural changes. Diffusion imaging is sensitive to subtle abnormalities to the brain microstructure, which serves as a proxy marker of tissue integrity. For instance, mean diffusivity (MD) tends to be increased in pathological tissue where the density of cellular membranes is reduced. In the presence of swelling or inflammation however, diffusivity decreases, something first witnessed in ischemic injury (Warach et al., 1995), but also in mild traumatic brain injury (Singh et al., 2016), in idiopathic inflammatory-demyelinating diseases (Canellas et al., 2007) or at a presymptomatic stage of familial Alzheimer's disease (Ryan et al., 2013). Fractional anisotropy (FA) is particularly sensitive to changes in tissue preferentially organised along one particular orientation, such as along axonal fibres, where it is usually found to be decreased in case of a pathological process as a result of the reduction in cellular boundaries that hinder diffusion. However in subcortical grey matter and regions of white matter crossing, where different orientational preferences can be observed, a selective degeneration of some of these connections can make this tissue appear less isotropic, leading to a (seemingly) counter-intuitive increase of FA (Ciccarelli et al., 2001; Douaud et al., 2009; Douaud et al., 2011).

As such, diffusion imaging has the potential to detect early brain tissue alterations in the presymptomatic stage preceding the volume loss detected at a macro-scale with structural MRI (Douaud et al., 2009; Rosas et al., 2010; Douaud et al., 2011). However, evidence for longitudinal microstructural changes in preHD is lacking so far. Previous longitudinal research did not reveal any microstructural MD or FA changes in preHD over a period of 18 months (Domínguez et al., 2013; Poudel et al., 2015), or 2 years (Odish et al., 2015b) compared with changes in healthy controls.

Here, we used diffusion MRI to investigate whether diffusion metrics could detect longitudinal changes over a one-year period in preHD, and discriminate across the different presymptomatic stages of the disease.

## 2 Methods

We investigated longitudinal changes in diffusion metrics in both grey matter (GM) and white matter (WM), comparing participants with presymptomatic HD (preHD) (*N* = 35) with age- and gender-matched healthy controls (CON)(*N* = 19). We used three complementary approaches. First, individual region-of-interest (ROI) analysis, inherently limited in its scope but unaffected by registration issues, to investigate longitudinal changes in MD and FA in the basal ganglia, the corpus callosum, the posterior limb of the internal capsule, and the cerebral peduncles. Second, probabilistic tractography was used to individually identify cortico-subcortical tracts (which cannot be isolated using anatomical ROIs) and to investigate longitudinal changes in diffusion tensor metrics in these tracts. Finally, tract-based spatial statistics (TBSS) was carried out as an unbiased, whole WM analysis approach to investigate changes in the entire WM skeleton of the participants.

### 2.1 Participants and study design

Participants were recruited from the Huntington's disease Centre of Excellence at the University of California, San Diego (Majid et al., 2011a; Majid et al., 2011b). They provided written informed consent in accordance with an institutional review board protocol of the University of California, San Diego. 38 preHD participants and 22 healthy CON were initially scanned on two visits, with a 1y interval between visits. Of these participants, three were excluded from the present data analysis due to missing or corrupted data, and three further subjects were excluded because the imaging protocol was not consistent with the one used for the other participants (different *b*-value, see 2. Data acquisition).

We therefore included 35 preHD participants and 19 age-, gender- and education-matched CON in the final diffusion data analyses (Table 1). The genetic expansion length of the mutant CAG repeat in HD is predictive for age at the onset of symptoms (Langbehn et al., 2010; Rosenblatt et al., 2012). To account for this variability, we further subdivided preHD participants into 3 different subgroups according to the number of years to the predicted onset of their disease: far from the onset of symptoms (FAR, *n* = 11), midway to the onset (MID, *n* = 11), and near the onset (NEAR, *n* = 13). The estimated age at onset was calculated using a prediction formula based on the length of each participant's CAG repeat, and the parental age at the onset of the disease (Aylward et al., 1996). Two of the participants in the NEAR subgroup progressed from presymptomatic to manifest stage of the disease between baseline and follow-up visits. We chose this formula, in particular over another one also commonly used (Langbehn et al., 2010), as this was the only one to accurately predict that these two participants who presented with manifest HD within one year should be in the group NEAR the onset of symptoms (**Supplementary Figure 1**).

**Table 1:** Demographic data for healthy controls and all preHD subjects, as well as for subgroups according to the onset of symptoms (FAR, MID, NEAR).

	Controls	PreHD	FAR	MID	NEAR
N	19	35	11	11	13
Sex	12F/7M	20F/15M	4F/7M	7F/4M	9F/4M
Age at baseline (y)	38.8 ± 12.1	41.8 ± 9.9	35.6 ± 6.1	38.8 ± 9.7	49.6 ± 7.8
Mutant CAG length range	–	41.9 ± 2.6 36–48	41.5 ± 3.2 36–47	43.1 ± 2.4 40–48	41.3 ± 1.8 39–45
Nonmutant CAG length range	–	18.3 ± 3.5 12 – 29	18.6 ± 2.3 16 – 24	18.6 ± 4.2 12 – 28	17.8 ± 3.9 12 – 29
Years of education	15.8 ± 2.5	15.6 ± 2.5	15.6 ± 2.6	15.5 ± 1.9	15.7 ± 2.9
UHDRS motor score at baseline	0.05 ± 0.2	1.7 ± 1.7	1.5 ± 1.6	1.5 ± 1.8	2.2 ± 1.9

All participants’ motor function was assessed using the Unified Huntington's Disease Rating Scale (UHDRS) motor, modified motor and confidence in motor scores. Cognitive ability was measured using the Mini–Mental State Examination (MMSE) and the Montreal Cognitive Assessment (MOCA), as well as the Wechsler Abbreviated Scale of Intelligence (WASI, matrix and vocabulary). All participants were also tested using the digit span test to assess their working memory, the symbol digit modality test, and the Stroop test, a cognitive interference task. Behavioural measures included the Symptom Checklist (SCL) 90, a self-report questionnaire assessing psychiatric symptoms (Derogatis and Unger, 2010), and the Barratt Impulsiveness Scale, a questionnaire designed to assess the personality construct (trait) of impulsiveness (Patton et al., 1995). All measurements were taken at both timepoints, except for UHDRS motor-related scores, which were assessed at baseline in CON.

### 2.2 Data acquisition

Data were acquired with a General Electric (Milwaukee, WI, USA) 1.5T EXCITE HDx scanner with an 8-channel phased-array head coil. Image acquisition included a high-resolution 3D T1-weighted inversion recovery spoiled gradient recalled (IRSPGR) sequence (echo time TE = 2.8 ms, repetition time TR = 6.5 ms, inversion time TI = 600 ms, flip angle = 12°, band width = 244 Hz/pixel, field of view = 24 cm, matrix size = 256 × 192, slice thickness = 1.2 mm).

Diffusion-weighted data were acquired using single-shot echoplanar imaging with isotropic 2.5 mm voxels, *b*-value = 1000s/mm² and 51 diffusion directions (TE = 80.4 msec, TR = 13,200 msec, field of view = 24 cm, matrix size = 96 × 96, 47 axial slices).

### 2.3 Data processing

We used the FMRIB software library (FSL, version 5.0.4) (Smith et al., 2004; Jenkinson et al., 2012) to pre-process the MRI data. First, diffusion-weighted and T1-weighted structural images were brain extracted (Smith, 2002). This was done in the halfway space (see more details on the registration procedure below in 3.4.) to avoid any bias in the brain extraction between any of the two timepoints. Brain-extracted T1 and FA maps were linearly registered to one another within-subject (12 degrees of freedom). A diffusion tensor model was fitted at each voxel of the brain-extracted data (Behrens et al., 2003), and MD and FA maps were created for each subject. Additional preprocessing steps were performed on the diffusion‐weighted images: head motion and within‐scan motion between scans were accounted for using a series of rigid body registrations, and image distortion caused by eddy currents was corrected in FSL. A probabilistic diffusion model with modelling of crossing fibres was fit within each voxel (Behrens et al., 2007), and probabilistic tractography was run in native space with default settings. We optimised pre-processing and image registration for the longitudinal study design to avoid any bias between timepoints (Thomas et al., 2009; Douaud et al., 2011).

#### 2.3.1 Grey matter region of interest analysis

The basal ganglia were chosen as *a priori* ROI for the GM analysis in this study, as these subcortical structures, and particularly the caudate nucleus and putamen, are the neuroanatomical substrates of neuropathological changes characteristic of HD (Furtado et al., 1996; Rosas et al., 2001; Vonsattel et al., 2011; Guo et al., 2012). An automatic segmentation tool, FIRST, was used on the T1-weighted images in their native space to delineate the caudate nucleus, putamen, globus pallidus, and nucleus accumbens, which we call “basal ganglia” in the following for convenience. The basal ganglia mask was subdivided into an anterior and posterior subdivision to reflect the structural connectivity profile of the basal ganglia – the more anterior part of the basal ganglia being predominantly connected to prefrontal areas and associated with cognitive and limbic processing, whereas the more posterior part is predominantly connected with sensorimotor regions of the brain (Lehericy et al., 2004; Draganski et al., 2008; Douaud et al., 2009). The subdivision was made along a coronal plane (in MNI space, *y* = 63). The binarized ROI masks were eroded in native space using a kernel with a sphere of radius 2 mm to ensure that the spatial extent of the seed masks was confined to the subcortical GM and to reduce partial volume effects. The arithmetic mean of the MD and FA values was then calculated across all voxels in these eroded ROI masks.

#### 2.3.2 White matter analysis: atlas-based ROIs

We used the corpus callosum (genu, body, splenium), the bilateral posterior limb of the internal capsule and cerebral peduncles as *a priori* ROIs from the JHU ICBM-DTI-81 WM atlas (Mori et al., 2008; Oishi et al., 2008), and the acoustic radiations from the Jülich histological probabilistic atlas as a negative control region (Burgel et al., 2006; Eickhoff et al., 2007). The posterior limb of the internal capsule and cerebral peduncles were chosen as ROIs as WM regions mainly containing fibres of the corticospinal tract, and showing longitudinal changes in diffusion metrics in early clinical HD (Della Nave et al., 2010; Gregory et al., 2015). The corpus callosum on the other hand was shown to play a crucial role in the pathophysiology of preHD (Bartzokis et al., 2007; Rosas et al., 2010; Di Paola et al., 2012). All ROI masks were eroded using a kernel with a sphere of radius 2 mm to avoid detecting signals from the GM or cerebrospinal fluid. The arithmetic mean of the MD and FA values was calculated across all voxels in the eroded ROIs.

#### 2.3.3 White matter analysis: cortico-subcortical ROIs using tractography

Briefly, we ran probabilistic tractography in native space using the same basal ganglia masks as defined for the GM ROI analysis as “seed” masks, i.e. where to start the tractography algorithm. In addition, we used simple, geometric target masks, as well as exclusion masks of the cerebrospinal fluid, brain stem and cerebellum, to focus the analysis on cortico-subcortical pathways (see below for more details). The weighted mean of the MD and FA values across all voxels belonging to each tract was calculated.

A probabilistic diffusion model was fit on the raw data, and crossing fibres were modelled within each voxel of the brain using the FSL tool, Bayesian Estimation of Diffusion Parameters Obtained using Sampling Techniques (BEDPOSTX, FSL default settings). We chose to estimate crossing fibres as it has been shown that upwards of 63% of the voxels is robustly estimated to contain more than one fibre population (Jeurissen et al., 2013). The output of BEDPOSTX was used to run probabilistic tractography in native space for each subject and for each time-point separately with seed, target, and exclusion masks using the FSL tool probtrackx2 (default settings). The basal ganglia masks used for the GM ROI analysis were used as seeds for probabilistic tractography. To focus the analysis on cortico-subcortical pathways, we used exclusion mask of the brainstem and cerebellum by drawing a rectangle in the axial plane at *z* = 33 covering the brainstem and cerebellum. Exclusion mask of the cerebrospinal fluid was created in native space by thresholding and binarizing the *S*_0_ image using an 85% threshold. Target masks, and exclusion masks of the brainstem and cerebellum were created in MNI standard space, and then registered onto each subjects’ FA image.

We did not attempt to reconstruct the cortico-spinal tract as it was covered both at the individual level by our ROIs in the posterior limb of the internal capsule and the cerebral peduncles, but also in these same regions by TBSS at the voxel-level. There are also well-known issues arising from using the entire, virtually-reconstructed tract to extract MD and FA values, such as averaging out opposite effects in regions of dominating vs. dominated fibres of interest (Groeschel et al., 2014).

We used the density of diffusion streamlines as weights to calculate the weighted mean of MD and FA across all voxels belonging to each tract, excluding those voxels in crossing fibre regions. In other words, a greater importance towards the contribution to the mean was assigned to voxels where many streamlines passed through, whereas a lesser weight was assigned to voxels were only few streamlines passed through. We defined voxels belonging to crossing fibre regions by thresholding (at 0.4) and binarizing the average FA image over the two time-points and all the subjects, this to principally exclude the voxels in the centrum semi-oval where three fibres cross (Douaud et al., 2011; Sotiropoulos et al., 2013), and to avoid averaging out opposite effects (Groeschel et al., 2014).

The resulting image was registered into each subject's native space using nonlinear transformation warps, and binarized to yield masks of crossing fibre regions.

#### 2.3.4 White matter analysis: whole white matter TBSS

The TBSS analysis was used to investigate whole-brain white matter microstructural abnormalities unconfounded by the choice of ROI. TBSS increases the sensitivity and the interpretability of the results by reducing registration error and partial volume effects compared with traditional voxel-wise approaches. This is important in a context of neurodegenerative disorders when there might be some substantial structural changes and hence cross-subject misalignments (Smith et al., 2006). We developed an optimised registration approach to register baseline and follow-up FA images in a study-specific template space, whereby the baseline and follow-up FA images were first registered into their halfway space for each subject. The average FA image between baseline and follow-up in halfway-space was then non-linearly registered onto the FMRIB FA template (FMRIB58). Both warps were concatenated and applied to create a study-specific template using an equal number of preHD participants and control subjects across both time-points. Finally, baseline and follow-up FA images for each subject were non-linearly registered to this study-specific template. The same spatial transformations were applied to the MD images. We then subtracted the baseline from the follow-up images to obtain a difference image of MD and FA for each subject. These difference maps were projected onto the TBSS skeleton to remove the effect of cross-subject spatial variability that remains after the nonlinear registration. Voxel-wise statistics were run using the smoothed (Gaussian kernel with sigma of 1.5 mm) skeletonised MD and FA difference images.

### 2.4 Statistical analyses

Statistical analyses of ROI-extracted imaging data, as well as clinical and behavioural measures were all carried out using Statistical Software Package for the Social Sciences (SPSS 20), except for the voxel-wise TBSS analyses which were carried out using ‘randomise’ in FSL. R (version 3.2.3) was used to create the longitudinal plots, and the PCA analysis was done in Python (using scikit-learn). Scripts are available at github.fmrib.ox.ac.uk/douaud/preHD.

#### 2.4.1 ROIs and tractography

ANOVA was performed on the 1y changes in diffusion tensor metrics (difference between the two timepoints) using SPSS for the preHD subgroups (according to estimated years to onset of symptoms) and healthy CON. Post-hoc tests, based on Tukey's honest significant difference (HSD) method, were used to test for significant pairwise group differences between FAR, MID, NEAR and CON. One-sample *t*-test was performed using SPSS to investigate the MD and FA difference between follow-up and baseline within each of the 4 groups (FAR, MID, NEAR and CON). Shapiro–Wilk test was used to test if the data, and the residuals from the ANOVA, were normally distributed. If the data were not normally distributed, non-parametric equivalent tests were used: Mann–Whitney U-test, Kruskal–Wallis ANOVA, and Dunn-Bonferroni test. All inferential statistical analyses were corrected for multiple comparisons using Bonferroni correction across ROIs, i.e. across the 2 basal ganglia GM ROIs, or the 6 WM ROIs.

#### 2.4.2 TBSS

For voxel-wise statistics, permutation-based non-parametric inference (5000 permutations) was used to test for changes in MD and FA between all preHD participants and healthy CON, and between CON and the 3 subgroups (Nichols and Holmes, 2002). Results were considered to be significant for *P* < 0.05, and corrected for multiple comparisons using threshold-free cluster enhancement with 2D optimisation (Smith and Nichols, 2009).

#### 2.4.3 Clinical and cognitive scores

We carried out one-sample *t*-test to assess the significant changes in all questionnaire scores in the entire preHD group (motor, cognitive and behavioural, see full break down in Fig. 4). We cross-correlated these (cross-sectional and longitudinal) scores for the entire preHD group to assess whether the 1y changes in those scores were consistent with the structure observed at each timepoint separately. In addition, a principal component analysis (PCA) was performed on these changes in all questionnaire scores to determine what the dominant 1y changes in these measures were. The changes in questionnaire scores were scaled before applying PCA using the ‘StandardScaler’ from scikit-learn (Python), so that these values had a zero mean and unit variance.

To make it possible to compare with our imaging results, we also performed ANOVA and post-hoc tests in a similar way as described above, i.e. across the 4 groups for all changes in cognitive and behavioural questionnaire scores (and 3 preHD sub-groups for the UHDRS motor-related scores). Finally, we investigated whether any of the significant diffusion changes observed across the four groups in the previous imaging analyses could be related to changes in motor, cognitive or behavioural changes.

Data were tested for normality, and if found non-normally distributed, equivalent statistical tests (described above) were used. All results were corrected for multiple comparisons across the number of questionnaires (*n* = 31).

## 3 Results

### 3.1 Grey matter region of interest analysis

**Fig. 1:**
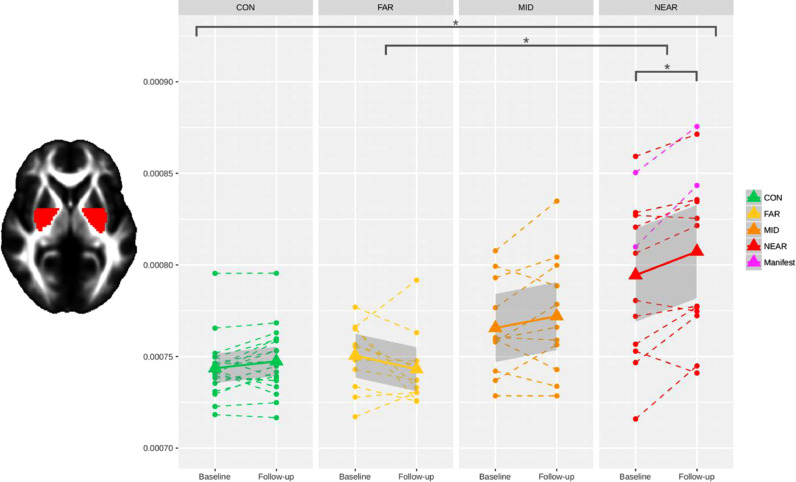
**Significant difference in longitudinal changes in MD across the four groups in the posterior basal ganglia**. Control group “CON”, preHD far from onset of symptoms “FAR”, midway to onset “MID”, and near the onset “NEAR”. Stars indicate significant longitudinal differences between groups and within groups, after adjusting for multiple comparisons across ROIs (Bonferroni correction). Each subject is represented by a dashed line, the group means by solid lines, with standard errors in shading.

Group comparisons using ANOVA revealed a statistically significant overall effect across the 4 groups (CON, FAR, MID, NEAR) on change in MD in the posterior basal ganglia (F_(3,50)_ = 4.5, *P* = 0.007, Fig. 1). Post-hoc pairwise group comparisons revealed a significant group difference in MD change in the posterior basal ganglia between FAR and NEAR (Cohen's

*d* = −3.63, *P* = 0.004). This effect was driven by a relative decrease in MD in FAR, compared with a relative increase in MD in NEAR. There was also a trend in the pairwise comparisons between FAR and MID (*P* = 0.094 before correction). Within the NEAR group, MD was significantly increased from baseline to follow-up in the posterior basal ganglia ROI. There was no statistically significant overall effect across the 4 groups on the FA change in the 2 ROIs of the basal ganglia.

### 3.2 White matter analysis

#### 3.2.1 Atlas-based ROIs

**Fig. 2:**
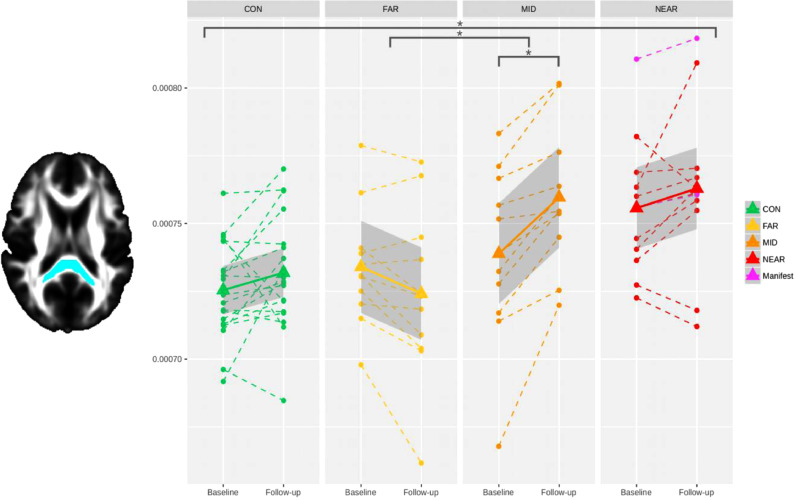
**Significant difference in longitudinal changes in MD across the four groups in the splenium of the corpus callosum**. Stars indicate significant longitudinal differences between groups and within groups, after adjusting for multiple comparisons across ROIs (Bonferroni correction). There was no outlier in any of the four groups.

We found a statistically significant overall effect of group (CON, FAR, MID, NEAR) on the MD change in the splenium of the corpus callosum (F_(3,50)_ = 6.7, *P* = 0.002, Fig. 2). Post-hoc pairwise group comparisons showed that this effect was driven by a significant group difference between FAR and MID (Cohen's *d* = −4.47, *P* = 0.00025), whereby MD showed a relative decrease in the FAR group compared with a relative, significant increase in the MID group. There was also a trend in the pairwise comparisons between CON and FAR, and FAR and NEAR (*P* = 0.048 and *P* = 0.057 before correction, respectively). There was no significant overall effect of group on the change in FA.

As expected, there was no significant effect of group in either diffusion metrics in the acoustic radiations.

#### 3.2.2 Cortico-subcortical tractography ROIs

We found no significant overall effect across groups or within groups on the change in MD or FA across the tracts connecting cortex and the basal ganglia as identified using probabilistic tractography (lowest *P* = 0.13, **see Supplementary Table 1**).

#### 3.2.3 Whole white matter voxel-wise analysis

There was no significant overall effect of group on MD change in the WM skeleton (lowest

*P* = 0.231).

**Fig. 3:**
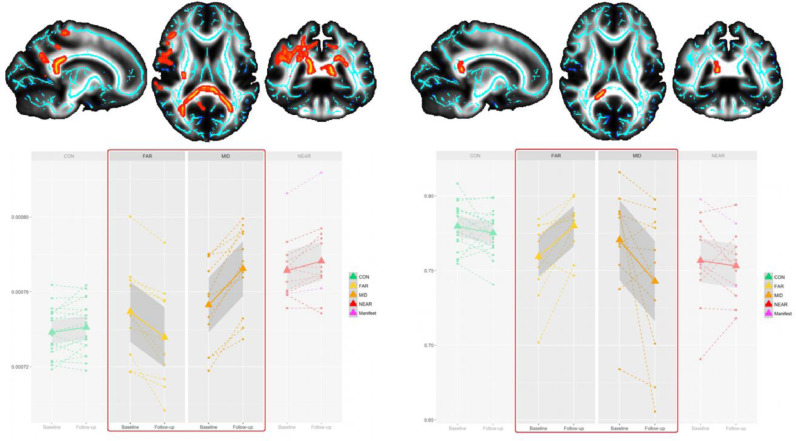
**Significant voxel-wise difference between FAR and MID in change in MD (left) and in FA (right).** Top, significant voxel-wise TBSS differences (dilated for visual purposes, red-yellow: 0.001 < *P*-corrected < 0.05) in MD and FA change between the preHD subgroups FAR and MID, overlaid on the TBSS skeleton (blue) and the FA study-specific template. Significant differences are found mainly in the splenium of the corpus callosum. Results are corrected for multiple comparisons across voxels using threshold-free cluster enhancement at *P* = 0.05. Bottom, plots showing for illustrative purposes the longitudinal change of the average MD values, as well as the FA values (opposite pattern to MD), over all significant voxels of the TBSS map for the contrast FAR vs. MID. The red frame indicates the significant effect found in TBSS. (For interpretation of the references to colour in this figure legend, the reader is referred to the web version of this article.)

Despite a lack of overall significant effect across the 4 groups, and in the interest of completeness, we still report here the results of pairwise group comparisons. These showed a significant group difference between FAR and MID mainly in the splenium of the corpus callosum, but also in the right parietal cingulum bundle, superior longitudinal fasciculus, posterior limb of the internal capsule and superior corona radiata (Fig. 3). This was again explained by a relative decrease of MD in FAR, and a relative increase in MID. Similarly, while there was no overall effect of group on FA, there was a significant group difference in change between FAR and MID in the right splenium of the corpus callosum, driven by a relative decrease in FA in MID, compared with a relative increase in FAR (Fig. 3).

There was an additional significant group difference in FA change between CON and MID in the right splenium extending into the posterior part of the body of the corpus callosum, superior longitudinal fasciculus and superior corona radiata, mainly explained by a decrease of FA in MID. Voxel-wise within group effect was found in MID, whereby MD was significantly increased and FA decreased mainly in the splenium of the corpus callosum, as well as the right superior longitudinal fasciculus (**Supplementary Figure 2**).

### 3.3 Relationship with motor, cognitive and behavioural measures

#### 3.3.1 Changes in questionnaire scores in the entire preHD group

We found a significant change in the entire preHD group in obsessive-compulsive (OC) symptoms on the Symptom Checklist-90 (SCL90, *P* = 3 × 10^−7^), but this decrease was mainly due to what appears to be strong test-retest effects. These test-retest effects were also exhibited in the CON group. As a result, there was no difference in changes in OC between the two CON and preHD groups. There was also a significant change in UHDRS modified motor score in the entire preHD group (*P* = 0.0012), and a trend in UHDRS confidence in motor score (*P* = 0.002 before correction).

**Fig. 4:**
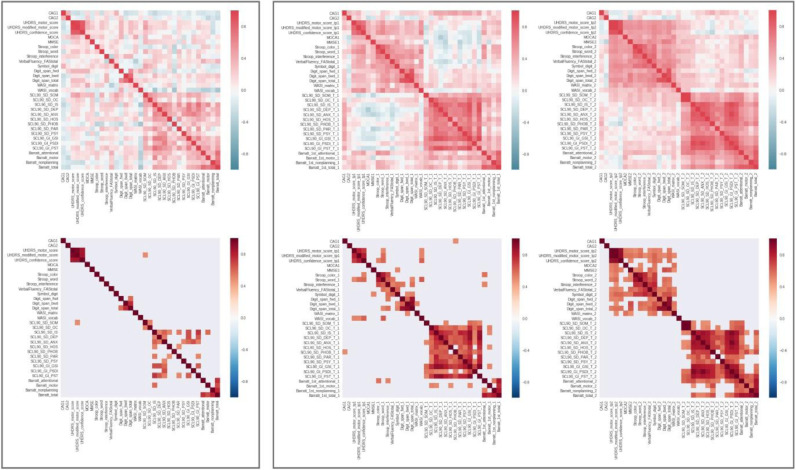
**Cross-correlation matrices between all questionnaire scores in the preHD group.** Top, all unthresholded correlations; bottom, thresholded at *P* = 0.05 corrected for multiple comparisons. Left, changes in these scores over a 1y period are weakly correlated in the preHD group. By contrast, these scores are highly correlated at both timepoints separately, baseline (middle) and follow-up (right). CAG1 = non-mutant length, CAG2 = mutant length.

In contrast with what could be observed at each separate timepoint, most of the changes in the preHD participants were only moderately correlated with one another (Fig. 4). This illustrates the highly variable rate of change in these scores across preHD participants. There were a few exceptions: the changes in UHDRS motor, modified motor and confidence in motor scores were highly correlated with one another (up to *r* = 0.8, i.e. 64% shared variance), and the changes in SCL90 interpersonal sensitivity were also moderately correlated with a range of other changes in SCL90 scores and in the motor scores on the Barratt Impulsiveness Scale (BIS).

**Fig. 5:**
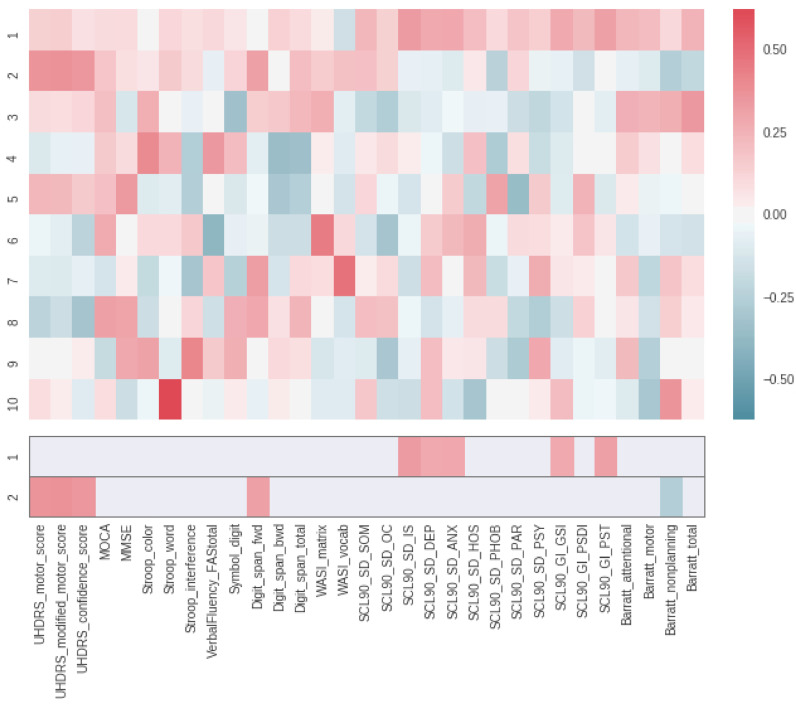
**Principal component analysis of the longitudinal changes in all questionnaire scores in the preHD group.** Top, first ten axes (unthresholded), which together explain more than 80% of the variance. Bottom, the first two axes, accounting for more than a third of the variance (35%), thresholded at 0.25 for visual purposes. While the first axis consisted of a linear combination of changes in the SCL90 behavioural scores, the second axis was mainly a linear combination of changes in the UHDRS motor-related scores.

A PCA in the preHD group further demonstrated that the main two orthogonal axes consisted of a linear combination of changes in the SCL90 scores, and a linear combination of changes in the UHDRS motor-related scores, respectively (the first 10 axes, explaining more than 80% of the variance of the data – see also **Supplementary Figure 3** – as well as the first 2 axes thresholded, are shown in Fig. 5).

#### 3.3.2 Group differences in changes in questionnaire scores

There was no significant difference across the 4 groups in changes in either cognitive or behavioural scores.

**Fig. 6:**
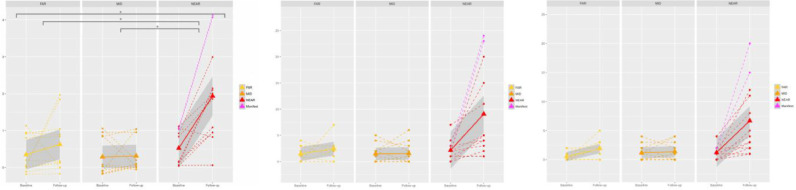
**Significant difference in longitudinal change in the UHDRS confidence in motor scores across preHD groups (left), and at trend level in the UHDRS motor and modified motor scores (middle, right).**Stars indicate significant longitudinal differences between groups and within groups, after adjusting for multiple comparisons across groups (Bonferroni correction). Difference in longitudinal change in UHDRS motor and modified motor scores across groups did not survive the correction (*P* = 0.004 and *P* = 0.003).

We found a significant effect across the 3 preHD subgroups (FAR, MID, NEAR) on the changes in UHDRS confidence in motor score (*P* = 0.0004), as well as a trend in UHDRS motor and modified motor score (*P* = 0.004 and *P* = 0.003 before correction)(Fig. 6). Post-hoc tests showed that this effect was driven by significant differences between FAR/MID and NEAR, as there was a strong, significant increase in the UHDRS motor, modified motor, and confidence in motor scores within the NEAR subgroup.

#### 3.3.3 Post-hoc correlation analyses

Finally, we wanted to investigate whether the significant diffusion changes observed across the four groups in the previous analyses (posterior basal ganglia, splenium of the corpus callosum) could be related to changes in motor, cognitive or behavioural changes. Despite the small sample sizes, we found that each group showed meaningful trends (*P* < 0.05) in correlations between changes in questionnaire scores and MD, even though this imaging measure showed both increase and decrease over time depending on the preclinical subgroup (**Supplementary Figure 4**). The strongest, significant effects were observed between changes in MD in the splenium of the corpus callosum and changes in phobia and hostility symptoms on the SCL90 for the MID group (Spearman's rho = +/−0.85, *P* = 0.0016 for both).

## 4 Discussion

Here we have investigated longitudinal changes over a one-year period in diffusion metrics in the GM and WM comparing healthy control subjects and preHD participants. We show that stratifying preHD participants into subgroups according to their estimated years to onset of symptoms yielded results revealing distinct patterns in the diffusion images at different presymptomatic stages. We found significant overall effects on MD change in the posterior basal ganglia and the splenium of the corpus callosum, as well as significant longitudinal differences in both diffusion metrics change between FAR and MID, FAR and NEAR, and CON and MID mainly in the splenium of the corpus callosum.

To the best of our knowledge, this is the first study that has demonstrated significant longitudinal changes in diffusion tensor metrics *within* and *between* the preclinical stages of HD. Previous longitudinal diffusion tensor MRI studies in preHD either did not detect any significant effects, or found significant effects only cross-sectionally (Domínguez et al., 2013; Odish et al., 2015b; Poudel et al., 2015), or solely when comparing patients with clinical HD to the preHD group (Domínguez et al., 2013; Poudel et al., 2015). One notable exception was that of Domínguez and colleagues’ (Domínguez et al., 2013), which found a significant within group effect, with an increase of MD in the caudate of preHD participants close to onset of symptoms (after similarly splitting them into two groups: close and far). Another study (Odish et al., 2015a), using network-derived measures of ‘nodal betweenness centrality’ on diffusion data, identified differences between their two preHD subgroups. In addition, previous diffusion MRI studies in clinical HD have also failed to detect any longitudinal effects (Vandenberghe et al., 2009; Sritharan et al., 2010). This most likely stems from the need to stratify participants with (preclinical) HD into meaningful subgroups, as finely as sample size allows, to ensure that increases and decreases in diffusion metrics are not averaged out across the various presymptomatic and clinical stages. This can be observed in Figs. 1**–**3, where no change in microstructure would have been found if all 3 preHD subgroups had been collapsed into one bigger group.

A previous study found a longitudinal reduction in MD in the putamen when comparing presymptomatic with manifest HD (Domínguez et al., 2013). This is in line with our results showing a decrease of MD at earlier stages of the disease, when compared with later stages (although our findings were obtained *within* the preclinical stages of the disease). Interestingly, in that study, as in ours, the pattern of change in diffusion metrics was also markedly different between posterior basal ganglia/putamen and anterior basal ganglia/caudate. There was a decrease then increase of MD (and the opposite pattern in FA) when transitioning towards later stages of the disease in the posterior basal ganglia/putamen, while there was a constant, progressive increase in MD in the anterior basal ganglia/caudate. This might indicate that the tissue of the anterior and posterior basal ganglia is undergoing a pathophysiological process that differs in timing.

Both the ROI and whole WM analyses revealed that changes in MD were mainly found in the corpus callosum. This is similar to previous research that found cross-sectional alterations in diffusion metrics in the corpus callosum across years-to-onset groups with presymptomatic HD (Rosas et al., 2010). These microstructural alterations preceded macrostructural atrophy, and were thought to reflect altered inter-hemispheric communication (Rosas et al., 2010). Notably, we did not find any significant longitudinal effect in the diffusion metrics over the cortico-subcortical tracts, confirming that the corpus callosum is a WM region particularly sensitive to very early damage in HD. This same region of the cerebral WM also shows pronounced longitudinal changes in the manifest stage of the disease (Poudel et al., 2015). In our study, the changes in diffusion metrics were mainly found in the splenium, rather than in the genu or body of the corpus callosum. This is in line with topologically selective cross-sectional alterations in diffusion metrics markedly found in the posterior corpus callosum in both presymptomatic and clinical HD(Rosas et al., 2010; Di Paola et al., 2012).

Besides being sensitive to longitudinal changes, an ideal state biomarker for preHD would shed light on the underlying pathophysiological mechanism of such changes. Overall significant differences in MD change in the posterior basal ganglia and splenium were mainly driven by decreases in MD in FAR compared with a relative increase in MID/NEAR. Such a decrease in MD is *prima facie* counter-intuitive in a neurodegenerative disorder, because increased MD is traditionally seen as a characteristic feature of neurodegeneration. However, in a diffusion study of preclinical Alzheimer's disease, MD was found significantly decreased at an early stage, and further simulation confirmed that an increase in microglia cell activation was sufficient to cause such a decrease at this early preclinical stage (Wang et al., 2015). The combination of axon damage, cell infiltration and oedema might cause the reversing of this effect on MD at a later stage. Similar findings have been shown in familial Alzheimer's disease, where MD was significantly decreased in the hippocampus and cingulum bundle at a presymptomatic stage, but increased at a symptomatic stage (Ryan et al., 2013). Microglial activation, a highly sensitive marker of neuronal insult (Kreutzberg, 1996), is known to occur in preHD (Tai et al., 2007) and pseudo-normalizes in later stages (Dowie et al., 2014). Taken together, this suggests that the initial decrease of MD consistently found at the earliest stage in our preHD cohort perhaps corresponds to neuro-inflammatory processes, and might thus represent very early microstructural sign of neurodegeneration. Ultimately however, only animal models or post-mortem cross-validation will be able to establish the underlying pathological process undergone by the brain tissue. Notably, despite MD showing both increase and decrease over time depending on the stage of the disease investigated, our imaging results in the grey and white matter correlated with changes in distinct clinical measures in every single presymptomatic group (albeit at a *P* < 0.05 trend level given the small sample size for each group, except for SCL90 phobia and hostility). The signs of these correlations also confirmed the deleterious nature of both the initial decrease of MD at the earliest stage of the disease, and its increase seen at later stages.

Unlike changes seen in structural MRI measures of subcortical and whole-brain volume in preHD (Majid et al., 2011a; Majid et al., 2011b) and further demonstrated over the course of 12, 24 and 36 months in the TRACK-HD study (Tabrizi et al., 2011; Tabrizi et al., 2012; Tabrizi et al., 2013), the behaviour of the changes in diffusion metrics is *non-monotonic*, showing different, meaningful pattern of increase and decrease for different preclinical stages of HD. We have shown here that these non-monotonic changes seen in diffusion MRI can be extremely helpful in understanding the underlying disease mechanisms, as well as monitoring progression towards the later stages of the disease and the effects of novel treatment options. These also helped finding significant differences between the preclinical stages of HD (FAR vs MID/NEAR). By contrast, only the UHDRS motor-related measures were able to distinguish preclinical stages thanks to a sharp increase in scores in NEAR (FAR/MID vs NEAR). In addition to clinical testing, structural and diffusion MRI should thus be considered together to combine sensitivity to volumetric changes and to the underlying pathological mechanisms.

One limitation of this longitudinal study in preHD is its small sample size but, as stated previously(Friston, 2012), as long as the necessary statistical precautions have been taken (here strict correction for multiple comparisons and non-parametric testing for non-normally distributed data), getting significant results with a small sample actually indicates that the effect is comparatively large (Cohen's d for differences between preclinical stages ranged from −3.6 to −4.5). We also ensured that our results showed the same effects when adding age, disease burden ((nCAG-35.5) × age) or sex in the statistical model as “nuisance” covariates. Despite an inherent loss of statistical power, all of our other results remained significant: our GM posterior basal ganglia after adding to the model either age (*F* = 4.3, *p* = 0.004), disease burden (*F* = 3.4, *p* = 0.015), or sex (*F* = 3.3, *p* = 0.018); and our original WM results in the corpus callosum after adding age (*F* = 5.2, *p* < 0.001), disease burden (*F* = 5.3, *p* < 0.001), or sex (*F* = 5.8, *p* < 0.001). Another potential limitation is in the diffusion imaging acquisition, which was carried out at 1.5T. We note however that diffusion tensor metrics in particular, as used here, are quite stable across different field strengths (Polders et al., 2011), and that the images benefitted from being acquired with isotropic voxels of 2.5 mm. Great care was also taken in the preprocessing of the data, as well as to account for partial voluming by using a combination of approaches (TBSS, and individual weighted probabilistic tractography and eroded ROIs). Finally, we re-run all analyses excluding the one preHD participant that had less than 39 CAG repeats (*n* = 36), as well as the two preHD participant who pheno-converted to symptomatic HD at the second timepoint. Albeit an inherent loss of power, all of the analyses yielded the same results.

In conclusion, diffusion MRI is able to detect significant and interpretable changes over a one-year period in preHD. Such short period of follow-up is crucial to establish whether a state biomarker has the potential to detect the effect of treatment. However, as Fig. 4 demonstrates, such changes are clinically less correlated and more difficult to track. To reveal these effects, it is necessary to divide subjects into clinically-meaningful subgroups according to their predicted age at onset of symptoms. The longitudinal decrease then increase in MD from earlier to later stages of the presymptomatic disease are perhaps the hallmarks of an initial early neuroinflammatory process, followed by neurodegeneration. Diffusion MRI thus offers key complementary information to the monotonic changes seen in structural MRI, and to clinical testing. In particular, we have shown here that diffusion metrics not only provide key mechanistic hypotheses about the underlying pathophysiological processes which could be tested using animal models or histological studies, but also much needed distinction between presymptomatic stages.

## Declaration of Competing Interest

None of the authors has any conflict of interest to declare.

## Appendix Supplementary materials

Image, application 1
