# Supplementary material for: One-year changes in brain microstructure differentiate preclinical Huntington's disease stages
Source: Neuroimage Clin. 2019 Dec 3;25:102099. doi: 10.1016/j.nicl.2019.102099 (PMC6931230; doi:10.1016/j.nicl.2019.102099)
Supplement: Supplementary file 1 [file mmc1.docx]

**Supplementary Figures**


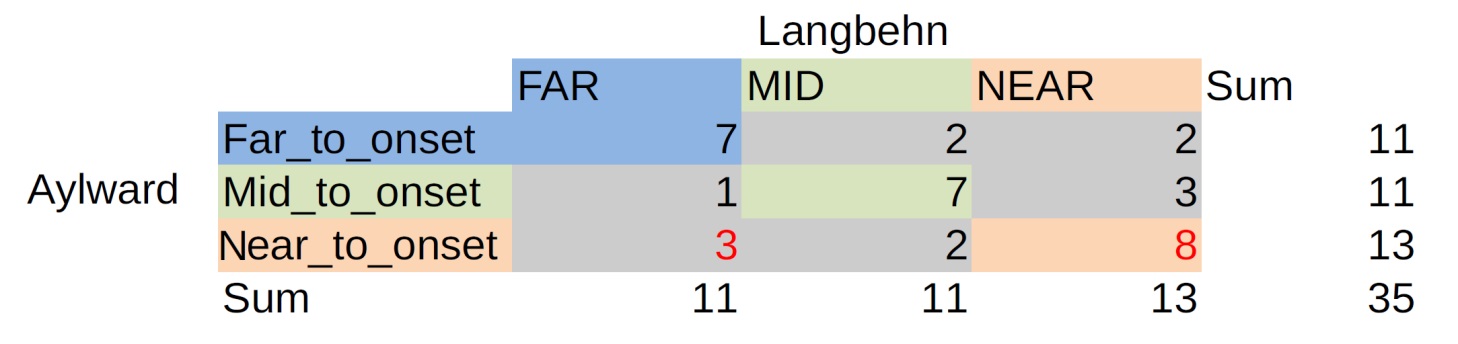


**Supplementary Figure 1: Contingency matrix for the classification of the HD carriers**. Rows present the break down based on the current formula from Aylward et al., 2010, columns based on the formula from Langbehn et al., 2010. In red are the groups where the two participants who developed manifest HD between baseline and follow-up one year later were classified. Please note that while both participants were in the near to onset group with the current formula, one of them was actually classified as being FAR from onset using the Langbehn formula.

# **
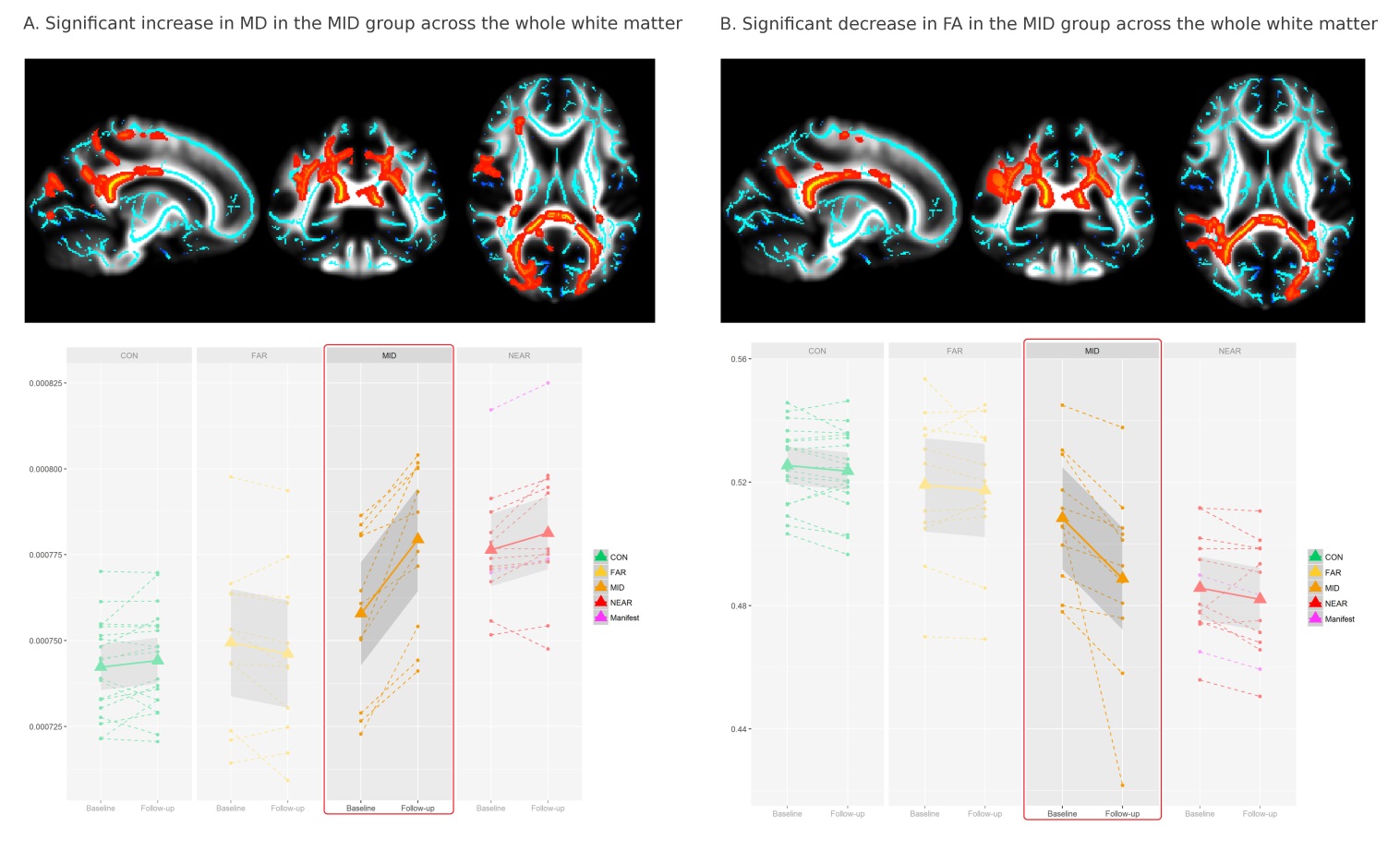
**

**Supplementary Figure 2**. **Significant longitudinal increase in mean diffusivity (MD) and fractional anisotropy (FA) within the group half-way to the onset of symptoms (MID).** **A.** Top, dilated TBSS map of the significant increase in MD (red-yellow) from baseline to follow-up in MID overlaid on the TBSS skeleton (blue) and the FA study specific template. Results at P < 0.05, corrected for multiple comparisons. Bottom, profile plots showing the longitudinal change of the average MD, over all significant voxels of the TBSS map for longitudinal increases in MID (as shown in A). The red frame indicates the significant effect found in TBSS. This plot is shown for illustrative purposes only. **B.** Same representation for the significant FA results.


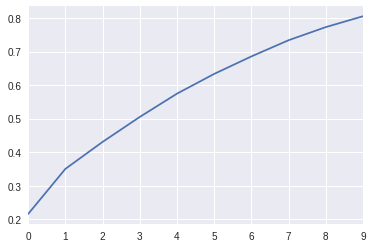


**Supplementary Figure 3: Cumulative variance explained by the first 10 PCs from the PCA on changes in cognitive and clinical scores**. The first 10 PCs explained more than 80% of the variance in those changes, while the first two PCs explained 35% of the variance.

**
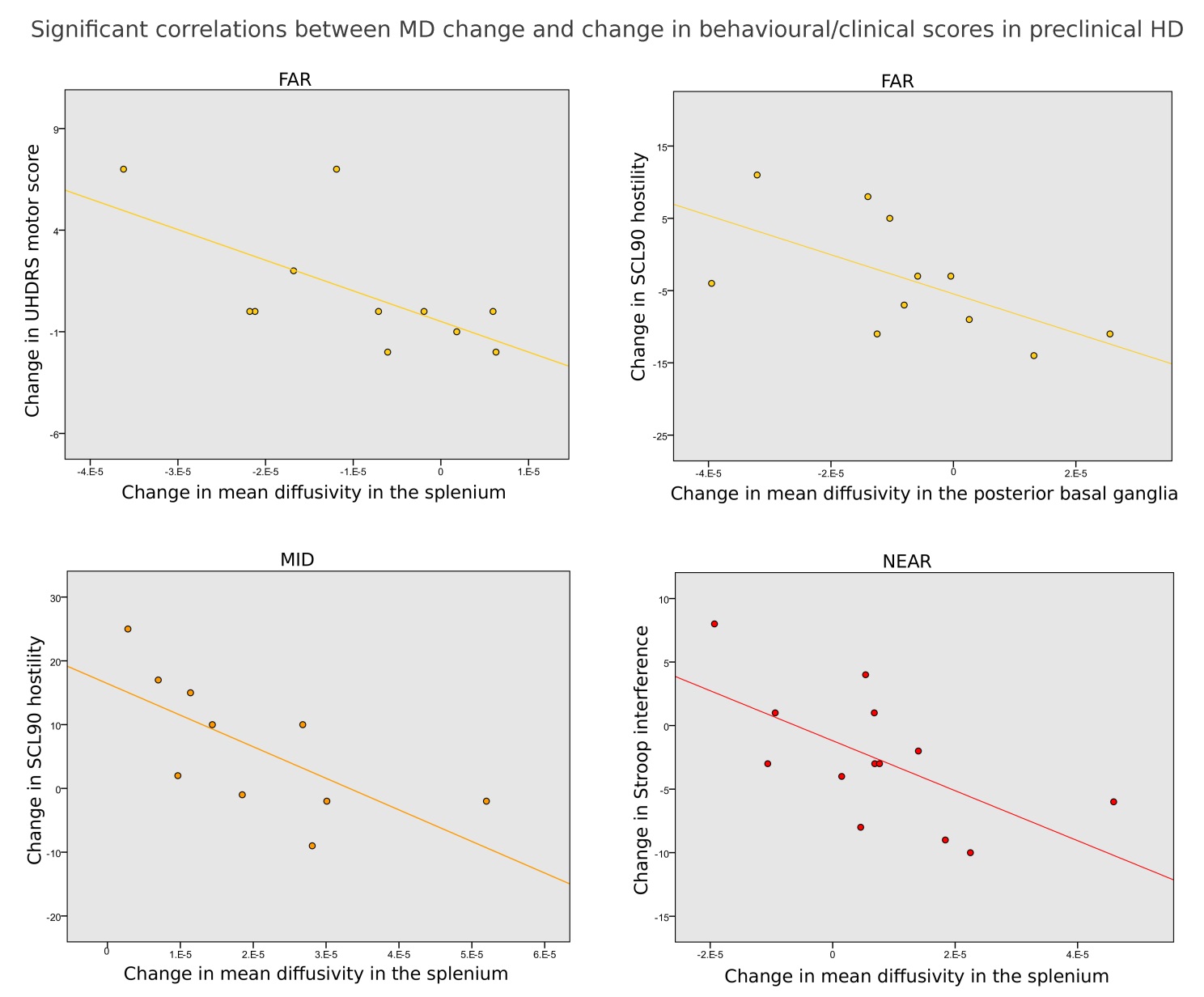
**

**Supplementary Figure 4.** **Correlations between mean diffusivity (MD) change and changes in behavioural/clinical scores**. Each preHD group shows a significant correlation (using Spearman’s rank correlation coefficient, not corrected for multiple comparisons). This includes the FAR group, demonstrating by the sign of the correlation that the decrease in MD is probably deleterious, and not compensatory (ρ = -0.66, *P* = 0.027, R^2^ = 0.40, top left; ρ = -0.61, *P* = 0.04, R^2^ = 0.37, top right), the MID group (ρ = -0.85, *P* = 0.002, R^2^ = 0.46, bottom left) and the FAR group (ρ = -0.57, *P* = 0.04, R^2^ = 0.39, bottom right).

**Supplementary Table**

|  | ROI | CON | | | FAR | | | MID | | | NEAR | | | ANOVA | |
| --- | --- | --- | --- | --- | --- | --- | --- | --- | --- | --- | --- | --- | --- | --- | --- |
|  |  | Mean (SD) | t(18) | *P* | Mean  (SD) | t(10) | *P* | Mean (SD) | t(10) | *P* | Mean (SD) | t(12) | *P* | F(3,50) | *P* |
| MD | Ant | 1.4  (1.3) | 0.05 | 0.96 | -7.4  (1.7) | -1.41 | 0.19 | -3.6 (8.2) | -1.45 | 0.18 | 4.4 (1.0) | 1.59 | 0.14 | 1.98 | 0.13 |
|  | Pos | -6.5  (1.8) | -0.15 | 0.88 | 5.5  (1.9) | 0.94 | 0.37 | 2.2  (1.2) | 0.58 | 0.57 | -2.3  (2.1) | -0.38 | 0.71 | 0.42 | 0.74 |
| FA | Ant | 2.8  (5.7) | 0.22 | 0.83 | -2.7  (7.7) | -1.14 | 0.28 | -1.5  (4.3) | -1.20 | 0.26 | 1.6  (3.9) | 1.50 | 0.16 | 1.43 | 0.24 |
|  | Pos | -9.4  (9.6) | -0.42 | 0.68 | 2.6  (9.5) | 0.91 | 0.38 | 4.9  (6.8) | 0.24 | 0.82 | -1.3  (1.0) | -0.44 | 0.67 | 0.44 | 0.72 |

**Supplementary Table 1.** **Longitudinal results from the probabilistic tractography analysis using anterior and posterior basal ganglia as seeds**. Mean change in mean diffusivity (MD) values and standard deviations (×10^-6^), and mean change in fractional anisotropy (FA) values and standard deviations (×10^-3^). P-values are uncorrected. There was no significant change within group, or between groups. ROI: region of interest, Ant: tracts reconstructed from the anterior basal ganglia, Pos: tracts reconstructed from the posterior basal ganglia. CON: control, FAR: far from the onset of symptoms, MID: mid-way from the onset of symptoms, NEAR: near the onset of symptoms.
